# Alterations of CD8^+^ T cells in the blood and salivary glands of patients with primary Sjögren’s syndrome

**DOI:** 10.1007/s10067-022-06491-7

**Published:** 2023-01-07

**Authors:** Hongxia Li, Yaxin Zhou, Pengyu Wang, Yafei Wang, Yuan Feng, Yan Zhang, Zhenbiao Wu

**Affiliations:** 1 Department of Rheumatology and Immunology, Air Force Medical Center, Air Force Medical University (Fourth Military Medical University), Beijing, China; 2 Department of Rheumatology and Immunology, Tangdu Hospital, Air Force Medical University (Fourth Military Medical University), 569 Xinsi Road, Xi’an, 710038 Shaanxi China

**Keywords:** CD8^+^ T cells, IFN-γ, Primary Sjögren’s syndrome, Salivary glands

## Abstract

**Objective:**

To identify the alterations of CD8^+^ T cells in blood and labial salivary glands (LSGs) of patients with Primary Sjögren’s syndrome (pSS).

**Methods:**

Blood samples from 24 pSS patients were assayed for CD38^+^ HLA-DR^+^ CD8^+^ (activated CD8^+^, aCD8^+^) T cells and serum IFN-γ and TNF-α, using flow cytometry and ELISA respectively, and compared with samples from 27 healthy controls. Immunohistochemistry was used to count CD8^+^ T cells in LSG tissues of 24 pSS patients and of 6 control patients with normal pathology.

**Results:**

pSS patients had more aCD8^+^ T cells than aCD4^+^ T cells (medians 33.13% vs*.* 9.43%, *p* < 0.0001), and had an increased level of aCD8^+^ T cells (medians 33.13% vs*.* 16.48%, *p* < 0.0001) and serum IFN-γ (medians 1026 pg/mL vs*.* 0.00 pg/mL, *p* < 0.0001) compared to the healthy controls. The levels of aCD8^+^ T cells and IFN-γ were both significant positively correlated with European League Against Rheumatism Sjögren’s Syndrome Disease Activity Index, IgG, anti-nuclear antibodies, rheumatoid factor. The LSGs focus score (FS) ≥1 group had more CD8^+^ T cell counts than 0≤ FS <1 group and control group (medians 256/mm^2^ vs*.* 126/mm^2^ and 256/mm^2^ vs*.* 64/mm^2^ respectively, both *p* < 0.05).

**Conclusion:**

The aCD8^+^ T cells and IFN-γ are positively correlated with each other, and predominantly elevated in the blood of pSS patients. In the LSG tissues of pSS, CD8^+^ T cell counts increase with severity of the lesions. CD8^+^ T cells may play crucial role in the pathogenesis of pSS.

**Table:** 

**Key Points** *• Primary Sjögren’s syndrome (pSS) is a chronic and systemic autoimmune disease. pSS patients had elevated blood levels of CD38* ^*+*^ *HLA-DR*^*+*^ *CD8*^*+*^ *T cells and IFN-γ.* *• The CD38* ^*+*^ *HLA-DR*^*+*^ *CD8*^*+*^ *T cells positively correlated with disease parameters and serum IFN-γ.* *• The salivary glands of pSS patients had appreciable CD8* ^*+*^ *lymphocyte infiltration. CD8*^*+*^ *T cells may play crucial role in the pathogenesis of pSS.*

**Supplementary Information:**

The online version contains supplementary material available at 10.1007/s10067-022-06491-7.

## Introduction

Primary Sjögren’s syndrome (pSS) is a chronic and systemic autoimmune disease characterized by dry eyes and dry mouth. Although this disease primarily targets the exocrine glands, it also affects extra-glandular systems and can lead to musculoskeletal, cutaneous, renal, pulmonary, and neurological manifestations [1].

The pathogenesis of pSS is complex and involves alterations in the innate and adaptive immune systems, including many immune cells, cytokines, and molecular markers [2]. Previous research on the immunopathogenesis of pSS have mainly focused on B cells and CD4^+^ T cells [2–5], but some other studies [6–9] showed that CD8^+^ T cells may also play a pivotal role. Therefore, it is necessary to investigate the abundances of CD8^+^ T cells in the blood and diseased tissues of patients with pSS. CD38^+^ and HLA-DR^+^ T cells were considered activated cells according to the Standardizing Immunophenotyping for the Human Immunology Project [10]. Some reports have showed increased levels of blood activated CD8^+^ T cells in pSS patients [8, 11], but they did not analyze the correlations between activated CD8^+^ T cells and multiple disease parameters. In this study, we analyzed CD38^+^ HLA-DR^+^ CD8^+^ (activated CD8^+^, aCD8^+^) T cells in the blood and its correlations with disease parameters of pSS patients. IFN-γ is necessary for activating Th1 cells which activate CD8^+^ T cells by secreting IFN-γ, and in return CD8^+^ T cells secret IFN-γ and TNF-α when activated. IFN-γ and TNF-α are strongly associated with aCD8^+^ T cells [7, 12], patients with SS had high levels of IFN-γ in serum [13] and T cells in labial salivary glands (LSGs) contained mRNA for IFN-γ [14], but its correlation with blood aCD8^+^ T cells in pSS has not been investigated to the best of our knowledge, so we also determined the serum levels of IFN-γ and TNF-α in these patients and examined its associations with aCD8^+^ T cells and multiple disease parameters.

Previous studies have shown the abundance of CD8^+^ T cells in diseased salivary gland tissues [7, 8], but whether the numbers of CD8^+^ T cells increase with the severity of the LSG lesions has not been studied. In this study, we analyzed CD8^+^ T cell counts in LSGs of pSS patients with different lesion severity and the correlations of CD8^+^ T cell counts with blood aCD8^+^ T cells, serum IFN-γ, and disease parameters. We also detected the expression of IFN-γ and TNF-α in CD8^+^ T cells in pSS LSG tissues using immunofluorescence.

## Materials and methods

### Patients

Twenty-four patents with pSS who received LSG biopsies at the First Affiliated Hospital of the Air Force Medical University (Xi’an, Shaanxi, China) between September 2020 and December 2020 were recruited. Diagnosis of pSS was according to the 2016 American College of Rheumatology/European League Against Rheumatism (ACR/EULAR) criteria [15]. Positive anti-SSA antibodies require testing for the anti-Ro60 antibodies; isolated anti-Ro52 antibodies are not specific for SS [1]. In the present study, anti-SSA antibodies refer only to anti-Ro60 antibodies and not to anti-Ro52 antibodies. Patients were excluded if they had any other connective tissue disease, sarcoidosis, amyloidosis, lymphoma, viral hepatitis, human immunodeficiency virus-positivity, a history of cervical irradiation, or if they had complications of infection or tumor. All samples were collected at the same time including salivary glands, blood, and clinical information. All patients were not taking any medications except for 2 patients, one taking 6mg/day of oral methylprednisolone and cyclosporine, another taking hydroxychloroquine combined with iguratimod, both less than 1 month which may not affect the results. But others who took moderate to high dose glucocorticoid or took immunosuppression more than 1 month were excluded because these might affect the number of activated CD8^+^ T cell in the blood. In addition, blood samples from 27 age- and sex-matched healthy controls (HCs) were used in the study. All participants provided written informed consent and all procedures were in accordance with the Declaration of Helsinki.

### Clinical assessments of primary Sjögren’s syndrome

The clinical data and medical records of pSS patients, including various clinical and laboratory parameters, were collected on the day of LSG biopsy and during follow-up. We used the EULAR Sjögren’s Syndrome Disease Activity Index (ESSDAI) evaluating the disease activity [16]. Unstimulated whole salivary flow (abnormal if ≤ 0.1ml/min in 15 min) [17] and Schirmer’s test (abnormal if ≤ 5 mm wetting of filter paper in 5 min) [18] were performed in most of patients.

### Laboratory and pathological determination

Anti-nuclear antibodies (ANA) were detected using an indirect immunofluorescence assay (EUROIMMUN, Lübeck, Germany), and anti-extractable nuclear antigen antibodies including anti-SSA, anti-SSB, and anti-Ro-52 antibodies were measured using immunodotting assay (EUROIMMUN, Lübeck, Germany). In this study, all pSS patients were ANA positive and ANA titres were graded at 4 levels (1:320, 1:1000, 1:3200, and 1:10000).

All patients underwent LSG biopsies. Hematoxylin and eosin (H&E) -stained sections were assessed by the same experienced pathologist, and pathology reports were recorded according to the Chisholm and Mason grading system (grade 0–4) [19]. The focus score (FS) which referred to the mean number of mononuclear cell infiltrates with 50 or more inflammatory cells per 4 mm^2^ of periductal or perivascular tissue [20] was recorded for each patient.

### Blood flow cytometry

Peripheral blood samples (2 mL) from the 24 pSS patients and 27 HCs were collected in heparin anticoagulant tubes on the day of LSG biopsy and subjected to flow cytometry on the same day. Supplementary Fig. 1 exemplifies the gating strategy and the fluorochromes. The cells were stained with a mixture of the following anti-human antibodies (Abs; all from BioLegend): anti-CD3 (300406), anti-CD4 (300530), anti-CD8α (301033), anti-CD38 (303506), and anti-HLA-DR (307610). After mixing, the cells were incubated at room temperature for 20 min in the dark, and 2 mL of red blood cell lysis solution was then added. After lysis at room temperature for 10 min in the dark and centrifugation, 2 mL of staining buffer (PBS) was added to the tubes, followed by two washes at 2000 rpm for 5 min. Flow cytometric analysis was performed using a DxFLEX Flow Cytometer (Beckman Coulter) and the corresponding included software.

### Detection of serum IFN-γ and TNF-α

The serum samples from the 24 pSS patients and 27 HCs were collected on the day of LSG biopsy and stored at −80°C until analysis by ELISA. The levels of IFN-γ and TNF-α were measured using kits from 4A Biotech according to the manufacturer’s protocols. The absorbance of each well was measured at 450 and 630 nm.

### Immunohistochemistry (IHC) and CD8^+^ T cell counts in LSG tissues

IHC was used to detect the expression of CD8 in the LSG tissues of the 24 pSS patients and of 6 control patients with normal pathology who only had symptoms of sicca, but no autoantibodies or any other disease. Paraffin embedded LSG tissues were sectioned (~4 μm) onto slides, deparaffinized at 60°C, and dehydrated using a series of xylene and alcohol baths. Epitope unmasking was performed using heat-induced antigen retrieval (Tris/EDTA, pH 9.0, 98–100°C for 15 min). The primary antibody was a mouse monoclonal antibody to CD8α (dilution 1:50, ab17147; Abcam). The chromogen was 3,3′-Diaminobenzidine (DAB) and the counterstain was hematoxylin.

IHC stained slides were converted into high-resolution digital sections using a digital pathological section scanner (PRECICE 500B, UNIC) and corresponding iScanner software. Then, each tissue section was evaluated by analysis of several sequential screenshots (10× magnification) of the entire section using the iViewer software (UNIC). The number of CD8^+^ T cells in each image was estimated using image analysis software (inForm 2.4.8, Akoya). The total number of CD8^+^ T cells divided by the total area was then used to calculate the number of CD8^+^ T cells per mm^2^. We divided the pSS patients into subgroups according to FS and analyzed the numbers of CD8^+^ T cells in LSG tissues.

### Detection of CD8, IFN-γ, and TNF-α in LSG tissues using immunofluorescence

The molecules of CD8, IFN-γ, and TNF-α were detected by immunofluorescence in the paraffin-embedded sections of LSG tissue. The same method was used as IHC described above, but fluorescence-conjugated secondary antibodies were used and DAPI was used as a chromatin counterstain. The primary antibodies were a mouse monoclonal antibody to CD8α (dilution 1:50, ab17147; Abcam), a rabbit monoclonal antibody to IFN-γ (0.02 mg/mL, MAB2853; Biotechne R&D Systems), and a goat polyclonal antibody to TNF-α (0.02 mg/mL, PA5-46945; Invitrogen). The secondary antibodies were goat anti-mouse IgG (FITC conjugated, dilution 1:100, Xi’an Zhuangzhi, China), goat anti-rabbit IgG (Cy3 conjugated, dilution 1:800, Xi’an Zhuangzhi, China), donkey anti-goat IgG with (Cy5 conjugated, dilution 1:2000, ab6566; Abcam). The stained slides were digitized with a Pannoramic scanner (3DHISTECH, Budapest, Hungary) and were analyzed by corresponding SlideViewer software.

### Statistical analysis

All statistical analyses were performed using the GraphPad Prism version 9.0. Continuous data are expressed as means ± standard deviation (SD) or median (interquartile range) as appropriate and comparisons were performed using the Mann-Whitney test. The correlations were determined using Spearman’s rank test. A *P-*value less than 0.05 was considered significant.

## Results

### Characteristics of patients with pSS

Twenty-four eligible patents with pSS who received LSG biopsies were included in this study (Table 1). The mean ages were 50.0 ± 14.9 years, and the male-to-female ratio was 1:23. The blood system was the most commonly involved (41.7%) and the median ESSDAI score was 7.6. Percentages of ANA titre ≥ 1:1000, anti-SSA antibody positivity, anti-Ro52 antibody positivity, and anti-SSB antibody positivity were 83.3%, 95.8%, 91.7%, and 41.7%, respectively.

**Table 1:** Characteristics of pSS patients and the healthy controls (HCs)

Characteristic	pSS (*n* = 24)	HCs (*n* = 27)
Age, years ± SD	50.0 ± 14.9	48.9 ± 15.8
Sex, male/female	1/23	1/27
Parotid gland enlargement, *n* (%)	4 (16.7)	-
Arthralgia, *n* (%)	5 (20.8)	-
Rash, *n* (%)	6 (25)	-
Blood system involvement, *n* (%)	10 (41.7)	-
Leukocytopenia, *n* (%)	6 (25)	-
Thrombocytopenia, *n* (%)	3 (12.5)	-
Hemolytic anemia, *n* (%)	1 (4.2)	-
Lung involvement, *n* (%)	3 (12.5)	-
Nerve involvement, *n* (%)	4 (16.7)	-
Kidney involvement, *n* (%)	0 (0)	-
Schirmer’s test ≤ 5 mm/5 min, *n* (%)	13/17 (76.5)	-
UWS flow rate ≤ 0.1 mL/min, *n* (%)	11/18 (61.1)	-
RF seropositive, *n* (%)	12/19 (63.2)	-
ANA seropositive, *n* (%)	24 (100)	-
ANA titre ≥ 1:1000, *n* (%)	20 (83.3)	-
Anti-SSA antibody seropositive, *n* (%)	23 (95.8)	-
Anti-Ro52 antibody seropositive, *n* (%)	22 (91.7)	-
Anti-SSB antibody seropositive, *n* (%)	10 (41.7)	-
IgG > 16 mg/L, *n* (%)	17/21 (81.0)	-
C3 < 80 mg/dL, *n* (%)	9/21 (42.6)	-
C4 < 16 mg/dL, *n* (%)	7/21 (33.3)	-
ESR > 20 mm/h, *n* (%)	9/18 (50)	-
ESSDAI ± SD	7.6 ± 4.2	-
LSG pathology		
FS ≥ 1, *n* (%)	12 (50)	
FS = 0, *n* (%)	6 (25)	
0 < FS < 1, *n* (%)	6 (25)	

*ANA*, anti-nuclear antibody; *ESR*, erythrocyte sedimentation rate; *ESSDAI*, EULAR Sjögren’s Syndrome Disease Activity Index; *FS*, focus score; *LSG*, labial salivary gland; *RF*, rheumatoid factor; *UWS*, unstimulated whole saliva

### Blood level of aCD8^+^ T cells and correlations with disease parameters

**Fig. 1:**
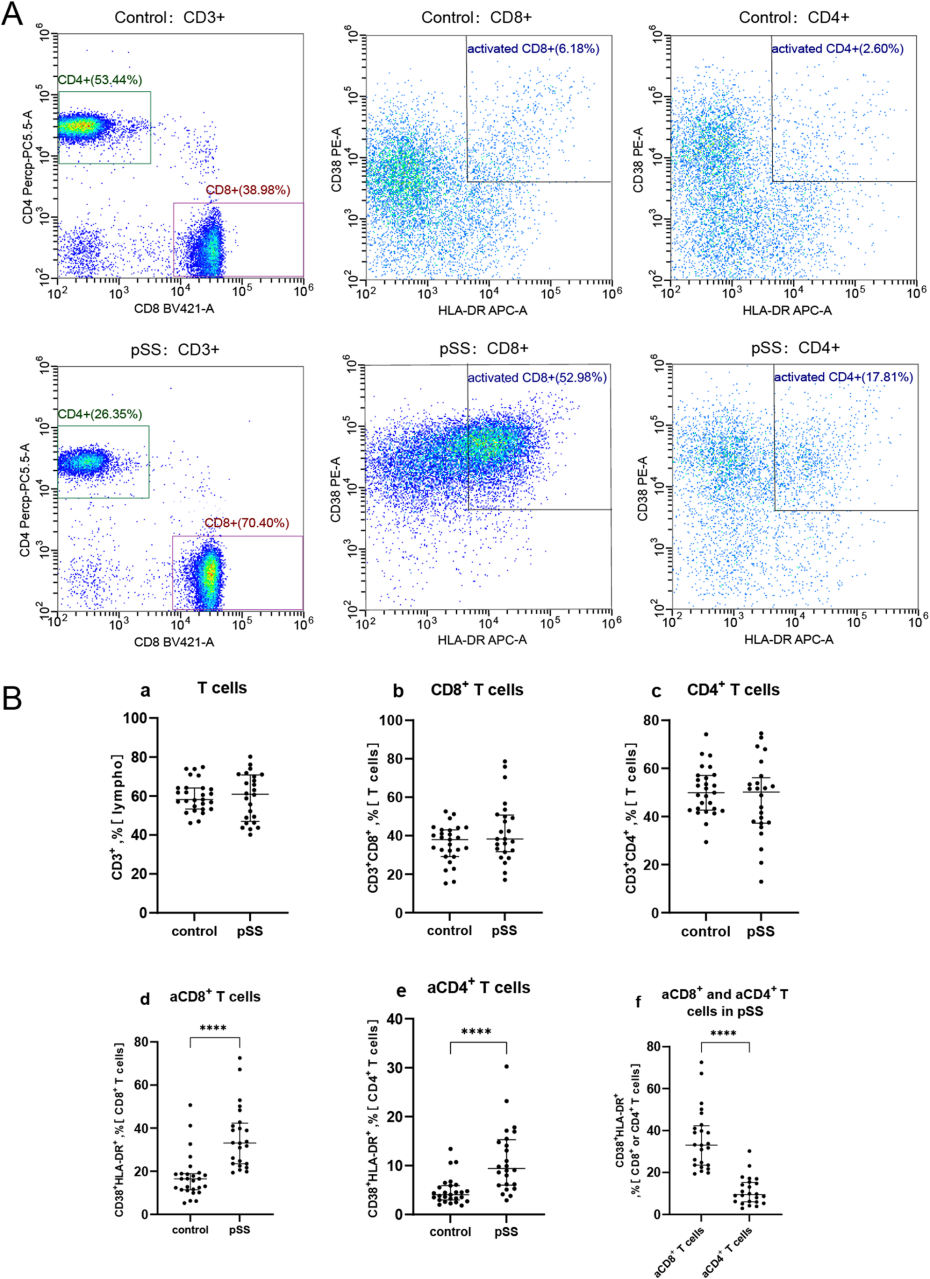
Blood level of aCD8^+^ T cells in pSS patients and the HCs. **A** Representative fluorescence-activated cell sorting images of aCD8^+^ and aCD4^+^T cells in blood samples of a control subject and a patient with pSS. **B** The HCs and patients with pSS had similar percentages of CD3^+^, CD4^+^, and CD8^+^ T cells (Ba-c), but the percentages of aCD8^+^ and aCD4^+^ T cells were significantly higher in the pSS patients than in the HCs (Bd-e). In pSS patients, the percentages of aCD8^+^ T cells was significantly higher than aCD4^+^ T cells (Bf). Horizontal lines represent median and interquartile range, ****p < 0.0001

We performed flow cytometry to determine the percentages of CD8^+^ and CD4^+^ T cells and their activated profiles in the blood samples of 24 patients with pSS and 27 HCs (Fig. 1A). The results indicated these two groups had similar percentages of CD3^+^, CD4^+^, and CD8^+^ T cells (Fig. 1Ba–c), but the percentages of aCD8^+^ and aCD4^+^ T cells were significantly higher in the pSS patients than in the HCs (medians 33.13% vs*.* 16.48% and 9.43% vs*.* 4.04% respectively, both *p* < 0.0001, Fig. 1Bd–e). In addition, separate analysis of pSS patients indicated they had a much higher percentage of aCD8^+^ T cells than aCD4^+^ T cells (medians 33.13% vs*.* 9.43%, *p* < 0.0001, Fig. 1Bf).

**Fig. 2:**
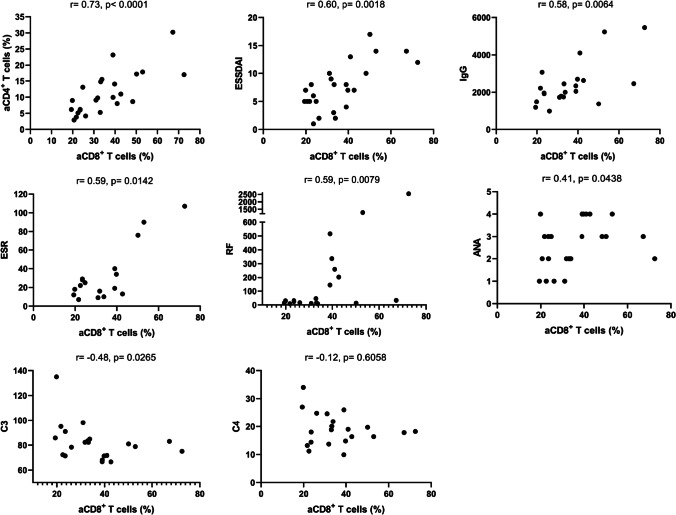
Correlations of blood aCD8^+^ T cells with disease parameters in pSS patients. Spearman correlation coefficients (*r*) and *p* values are indicated. Activated CD8^+^ T cells had significant positive correlations with aCD4^+^ T cells, ESSDAI, IgG, ESR, RF, and ANA, and had a negative correlation with complement C3 in pSS patients. IgG: mg/dL; ESR: mm/h; RF: IU/mL; C3: mg/dL; C4: mg/dL. ANA titres graded at 4 levels (1:320, 1:1000, 1:3200, and 1:10000)

Because aCD8^+^ T cells are predominant in the blood of pSS patients, we determined the correlation of aCD8^+^ T cells with aCD4^+^ T cells and disease parameters of pSS (Fig. 2). The results showed that aCD8^+^ T cells had significant positive correlations with aCD4^+^ T cells (*r* = 0.73, *p* < 0.0001), ESSDAI (*r* = 0.60, *p* = 0.0018), IgG (*r* = 0.58, *p* = 0.0064), erythrocyte sedimentation rate (ESR; *r* = 0.59, *p* = 0.0142), rheumatoid factor (RF; 0.59, *p* = 0.0079), and ANA (*r* = 0.41, *p* = 0.0438); a negative correlation with complement C3 (*r* = −0.48, *p* = 0.0265); and no significant correlation with complement C4 (*r* = −0.12, *p* = 0.6058). In contrast, aCD4^+^ T cells only had a positive correlation with ESSDAI (data not shown) among above indicators. These results suggest that aCD8^+^ T cells may function in the pathogenesis of pSS.

### Serum IFN-γ and TNF-α and correlations with blood activated T cells and disease parameters

**Fig. 3:**
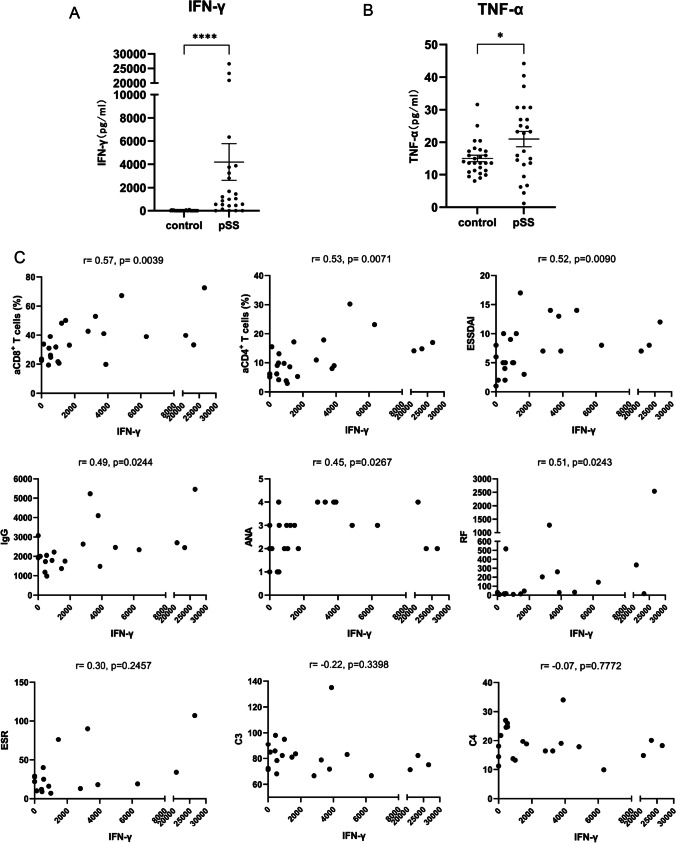
Serum levels of IFN-γ and TNF-α in pSS patients and the HCs, and the correlations of the IFN-γ with disease parameters. **A** Serum levels of IFN-γ in pSS patients and the HCs. **B** Serum levels of TNF-α in pSS patients and the HCs. **C** The correlations of IFN-γ with disease parameters in pSS patients. Spearman correlation coefficients (*r*) and *p* values are indicated. IFN-γ level had significant positive correlations with aCD8^+^ T cells, aCD4^+^ T cells, ESSDAI, IgG, ANA, and RF. Horizontal lines represent median and interquartile range, *p < 0.05, ****p < 0.0001. IFN-γ: pg/mL; IgG: mg/dL; ESR: mm/h; RF: IU/mL; C3: mg/dL; C4: mg/dL. ANA titres graded at 4 levels (1:320, 1:1000, 1:3200, and 1:10000)

IFN-γ and TNF-α are closely associated with the functions of CD8^+^ T cells. Therefore, we used ELISA to measure the serum levels of these cytokines in 24 pSS patients and 27 HCs (Fig. 3A–B). The level of IFN-γ was much greater in pSS patients (medians 1026 pg/mL vs*.* 0.00 pg/mL, *p* < 0.0001). The level of TNF-α was also significantly higher in pSS patients (medians 23.04 pg/mL vs*.* 13.57 pg/mL, *p* = 0.0494), although this difference was not as great as IFN-γ. These results suggested that these two cytokines—especially IFN-γ—might play crucial roles in the disease process of pSS.

Next, we assessed the correlations of the level of IFN-γ with different activated T cells and disease parameters (Fig. 3C). The results indicated that the IFN-γ level had significant positive correlations with aCD8^+^ T cells (*r* = 0.57, *p* = 0.0039), aCD4^+^ T cells (*r* = 0.53, *p* = 0.0071), ESSDAI (*r* = 0.52, *p* = 0.0090), IgG (*r* = 0.49, *p* = 0.0244), ANA (*r* = 0.45, *p* = 0.0267), and RF (*r* = 0.51, *p* = 0.0243); although there were no significant correlations with complement C3, complement C4, or ESR. In contrast, TNF-α had no significant correlations with any of these variables (data not shown). These results demonstrated that an increased IFN-γ level was associated with an elevated level of aCD8^+^ T cells and multiple indicators of pSS.

### CD8^+^ T cell counts in LSGs and correlations with blood activated T cells, serum IFN-γ, and disease parameters

**Fig. 4:**
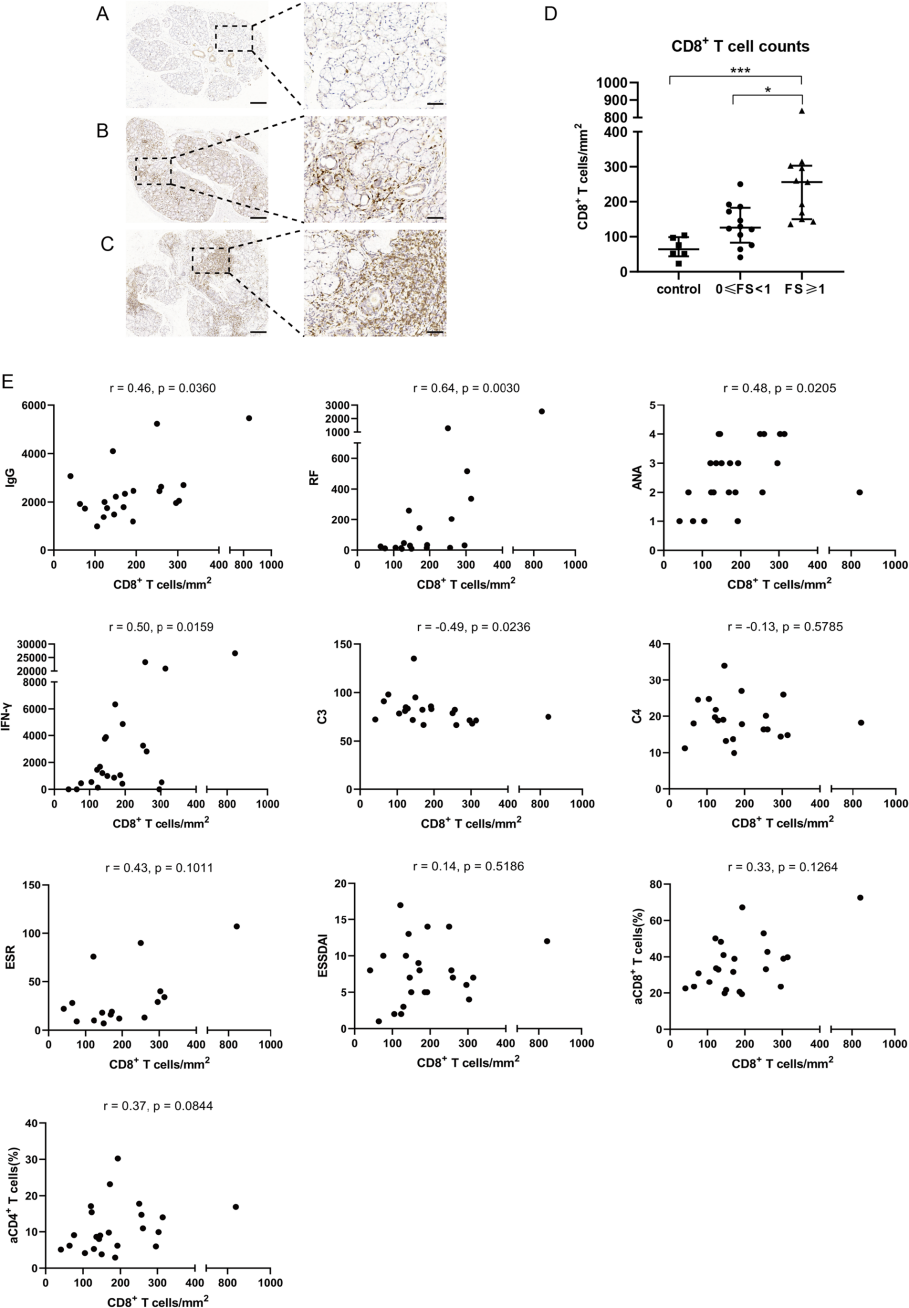
CD8^+^ T cell counts in LSG tissues and its correlations with disease parameters. **A**–**C** Representative images of three groups of patients: control patients (*n* = 6, **A**), pSS patients who were salivary gland 0≤ FS <1 (*n* = 12, **B**), and pSS patients who were salivary gland FS ≥1 (*n* = 11, **C**). **D** Comparations between the three groups. **E** Correlations of CD8^+^ T cell counts with disease parameters in pSS patients. Spearman correlation coefficients (*r*) and *p* values are indicated. CD8^+^ T cell counts had significant positive correlations with IgG, RF, ANA, and IFN-γ and had a negative correlation with complement C3. Left scale bars = 200 μm, right scale bars = 50 μm. Horizontal lines represent median and interquartile range, *p < 0.05, ***p < 0.001. IFN-γ: pg/mL; IgG: mg/dL; ESR: mm/h; RF: IU/mL; C3: mg/dL; C4: mg/dL. ANA titres graded at 4 levels (1:320, 1:1000, 1:3200, and 1:10000)

We used IHC to analyze CD8^+^ T cell counts by calculating the number of CD8^+^ T cells per mm^2^ in tissue sections of 23 pSS patients and 6 control patients. One of the 24 patients had insufficient LSG tissue for pathological analysis, so we divided the remaining 23 pSS patients into salivary gland 0≤ FS <1 group (*n* = 12) and salivary gland FS ≥1 group (*n* = 11). We stained these cells with a monoclonal anti-human CD8α antibody (Fig. 4A–C). These results demonstrated that the LSGs FS ≥1 group had more CD8^+^ T cell counts than 0≤ FS <1 group and control group (medians 256/mm^2^ vs*.* 126/mm^2^ and 256/mm^2^ vs*.* 64/mm^2^ respectively, both *p* < 0.05, Fig. 4D).

Furthermore, we determined the correlation of CD8^+^ T cell counts with serum IFN-γ and disease parameters of pSS (Fig. 4E). The results indicated that CD8^+^ T cell counts had significant positive correlations with IgG (*r* = 0.46, *p* = 0.0360), RF (*r* = 0.64, *p* = 0.0030), ANA (*r* = 0.48, *p* = 0.0205), and IFN-γ (*r* = 0.50, *p* = 0.0159); a negative correlation with complement C3 (*r* = −0.49, *p* = 0.0236); and no significant correlation with other variables.

### The expression of IFN-γ and TNF-α in CD8^+^ T cells in LSGs

**Fig. 5:**
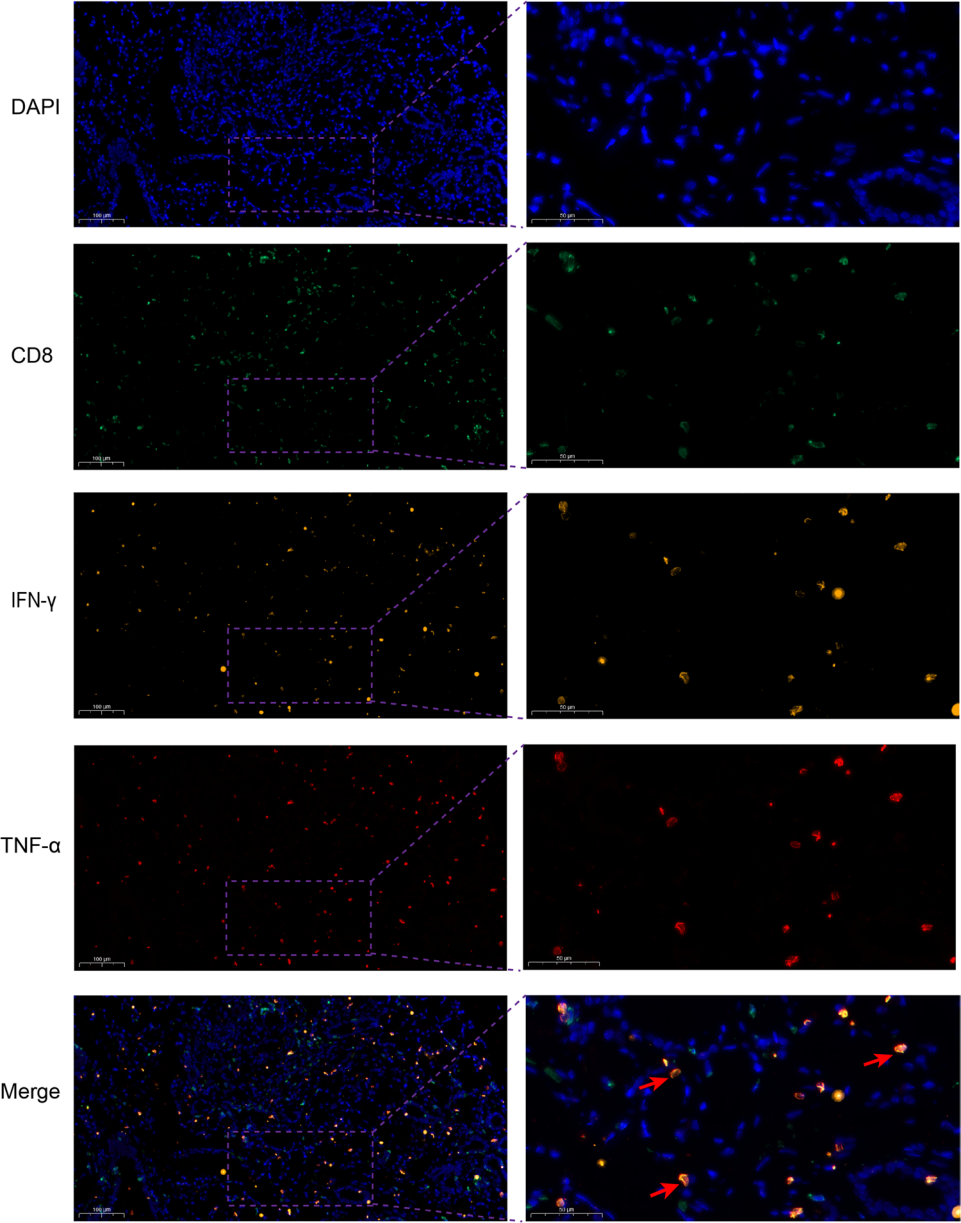
The expression of CD8, IFN-γ, and TNF-α in LSG tissues. The molecules of CD8 (green), IFN-γ (orange), and TNF-α (red) were detected by immunofluorescence in the paraffin-embedded sections of LSG tissue of a pSS patient with focus score 2.9, and DAPI was used to counterstain the nuclei. The red arrows show CD8^+^ T cells with positive IFN-γ and positive TNF-α in the Merge picture. Left scale bars = 100 μm, right scale bars = 50 μm

We analyzed the expression of IFN-γ (orange) and TNF-α (red) in CD8^+^ (green) T cells using immunofluorescence in the LSG tissues (as shown in Fig. 5). The results showed that the LSG tissues of pSS patients had positive expression of IFN-γ and TNF-α which almost were simultaneously expressed in the CD8^+^ T cells. Approximately half of CD8^+^ T cells in the domain of non-lymphocytic foci expressed IFN-γ and TNF-α, but in lymphocytic foci, only a small proportion of CD8^+^ T cells were positive (as shown in Supplementary Fig. 2).

## Discussion

Many researchers have focused on the role of alterations in B cells in the pathogenesis of pSS based on clinical observations, the presence of serum autoantibodies, and hypergammaglobulinemia [3]. Similarly, 83.3% of our patients had high titres of ANA (≥ 1:1000) and 81% had high levels of IgG (> 16 mg/dL). However, apart from their known role in congenital heart block, these autoantibodies do not seem to play a direct role in the pathogenesis of pSS [2]. In this study, we investigated the distributions of CD8^+^ T cells in the blood and LSG tissues of pSS patients and their associated cytokines (IFN-γ and TNF-α), and further analyzed their correlations with multiple indicators of disease status.

HLA-DR and CD38 are markers indicative of activated T lymphocytes [10]. Our analysis demonstrated pSS patients had a higher percentage of aCD8^+^ T cells than aCD4^+^ T cells, and that the percentage of aCD8^+^ T cells positively correlated with aCD4^+^ T cells and clinical parameters (ESSDIA, IgG, ANA, RF, and ESR). In contrast, aCD4^+^ T cells only had a significant positive correlation with ESSDAI. This demonstrated that aCD8^+^ T cells were predominant among activated T cells in the blood of pSS patients and may play an important role in the pathogenesis of this disease.

IFN-γ has important functions in immune responses to pathogens and tumors, and aberrant IFN-γ expression is associated with a number of autoimmune diseases, including pSS [21, 22]. This cytokine is mainly produced by CD4^+^ Th1 cells, CD8^+^ cytotoxic T lymphocytes (CTLs), and natural killer cells [23]. IFN-γ is essential for the differentiation of CD8^+^ T cells into CTLs, and CTLs can also secrete IFN-γ, a positive feedback response [12]. Therefore, there is a close association between IFN-γ and CD8^+^ T cells. In this study, we measured the serum levels of IFN-γ and TNF-α in pSS patients. The results demonstrated that the level of serum IFN-γ was much higher in pSS than in HCs, and was positively correlated with aCD8^+^ T cells, aCD4^+^ T cells, ESSDIA, IgG, RF, and ANA. In contrast, the TNF-α level was not correlated with any of these parameters, although it was marginally higher in pSS patients than HCs. We conclude that the blood levels of aCD8^+^ T cells, aCD4^+^ T cells, and IFN-γ were positively correlated in patients with pSS, and they all were associated with ESSDIA and the production of autoantibodies. In addition, we also investiged the expression of IFN-γ and TNF-α in LSG tissues, showing that IFN-γ and TNF-α were expressed almost exclusively in CD8^+^ T cells in pSS patients, and that only a few CD8^+^ T cells in lymphocytic foci expressed IFN-γ and TNF-α. Therefore, we speculate that aCD8^+^ T cells may exist near acini but not in lymphocytic foci, and CD4^+^ T cells may not express IFN-γ and TNF-α in LSG tissues of pSS patients, which need to be confirmed by further study.

Previous literature suggests the possible reason for the increased levels of aCD8^+^ T cells and IFN-γ and their correlations with elevated IgG and ANA in pSS [24, 25]. The pSS patients have an increased level of B-cell activating factor (BAFF) in serum and in salivary gland tissues, and IFN-γ is one of the major cytokines that promotes the secretion of BAFF [24]. Thus, an increased IFN-γ level stimulates the secretion of BAFF, and this facilitates the activation and proliferation of autoimmune B cells which then secrete autoantibodies [3, 25]. In general, autoreactive B cells and T cells communicate with each other via cytokines, chemokines, and other immune molecules.

We also stained CD8^+^ T cells using immunohistochemistry in LSG tissue sections of pSS patients in order to understand the number of infiltrated CD8^+^ T cells. The results showed that the LSG tissues of pSS patients had appreciable CD8^+^ lymphocyte infiltration and the numbers of CD8^+^ T cells increased with the severity of the LSG lesions suggesting that CD8^+^ T cells may contribute to damage of salivary glands rather than bystanders. Interestingly, we found that the CD8^+^ T cells counts had significant positive correlations with IgG, RF, ANA, and IFN-γ, indicating that CD8^+^ T cells in diseased LSGs may act indirectly on B cells by secreting IFN-γ.

There are some limitations in this study. Relatively small number of patients were included in this study. In addition, we did not investigate the cytokine expression of IFN-γ and TNF-α in peripheral blood CD8^+^ T cells, and did not reveal the specific mechanisms of CD8^+^ T cells in the pathogenesis of pSS. However, we have planned to do such job and will explore how the cells of LSGs were damaged by CTLs, hoping to provide some insights for pSS in the future.

In conclusion, our study indicated that pSS patients have an obviously increased blood level of aCD8^+^ T cells which are positively correlated with the serum levels of IFN-γ and multiple disease parameters. In LSG tissues of pSS, CD8^+^ T cell counts increase as the severity of the LSG lesions increased. These results suggest that CD8^+^ T cells may play crucial role in the pathogenesis of pSS.

## Supplementary Information

Supplementary Fig. 1 **Different gating strategies used for blood flow cytometry.** Lymphocytes were in P1 gate (A), T cells were in CD3+ gate (B), CD4^+^ T cells and CD8^+^ T cells were in CD3+CD4+ and CD3+CD8+ gates respectively (C), and CD38+ and HLA-DR+ T cells were considered activated T cells (D and E). (PNG 499 kb)

High Resolution Image (TIF 71607 kb)

Supplementary Fig. 2 **The expression of CD8, IFN-γ and TNF-α in lymphocytic foci of LSG tissues.** The molecules of CD8 (green), IFN-γ (orange) and TNF-α (red) were detected by immunofluorescence in the paraffin-embedded sections of LSG tissue of a pSS patient with focus score 2.9, and DAPI was used to counterstain the nuclei. Right magnified pictures show only a few CD8^+^ T cells of lymphocytic foci were positive for IFN-γ and TNF-α which are directed by red arrows in the Merge picture. Left scale bars = 100 μm, right scale bars = 50 μm. (PNG 2695 kb)

High Resolution Image (TIF 22841 kb)
