# Supplementary figures and images for: Alterations of CD8+ T cells in the blood and salivary glands of patients with primary Sjögren’s syndrome
Source: Clin Rheumatol. 2023 Jan 7;42(5):1327–38. doi: 10.1007/s10067-022-06491-7 (PMC10102090; doi:10.1007/s10067-022-06491-7)

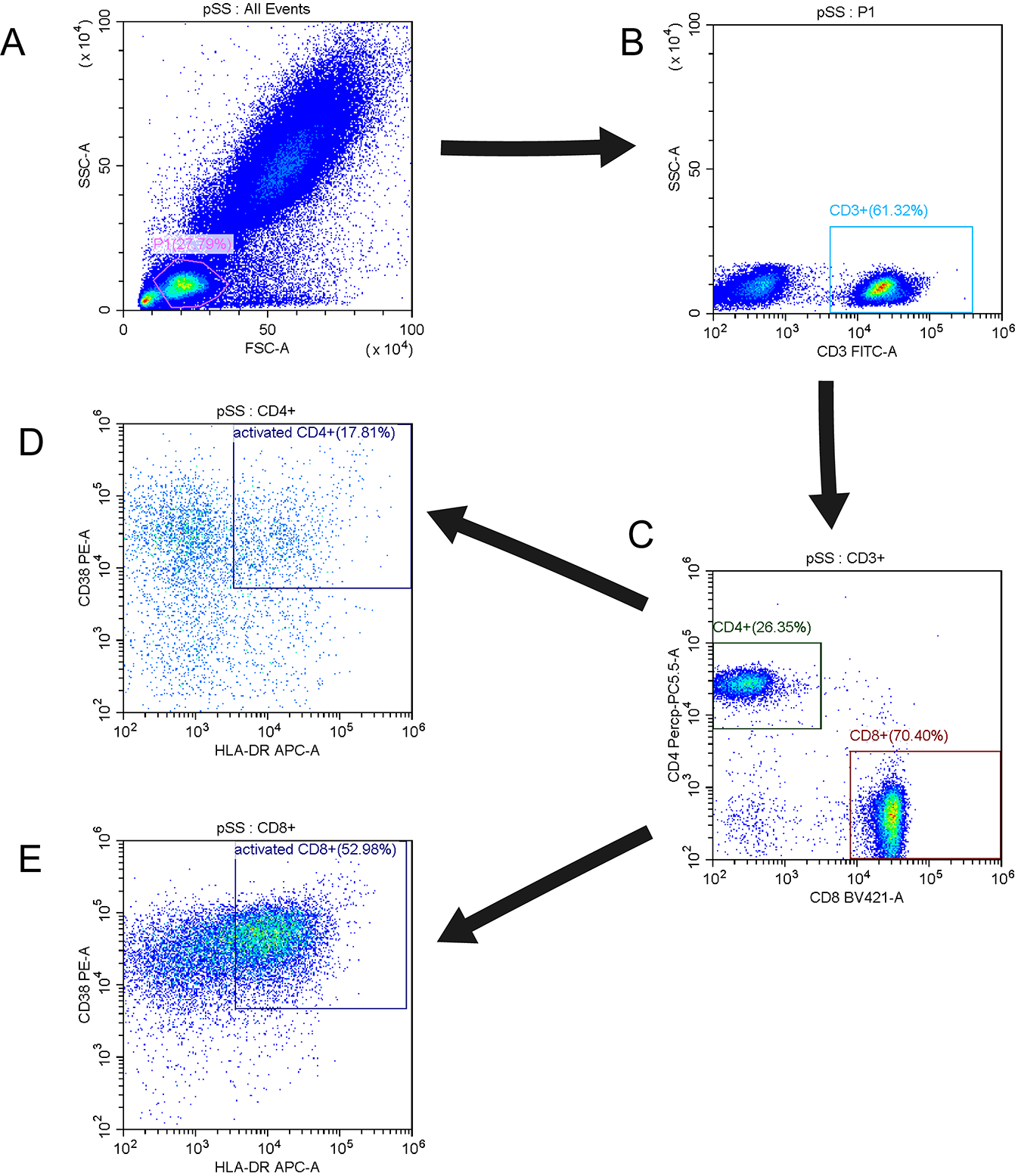

Supplement: Supplementary file 1 — Different gating strategies used for blood flow cytometry. Lymphocytes were in P1 gate (A), T cells were in CD3+ gate (B), CD4+ T cells and CD8+ T cells were in CD3+CD4+ and CD3+CD8+ gates respectively (C), and CD38+ and HLA-DR+ T cells were considered activated T cells (D and E). (PNG 499 kb) [file 10067_2022_6491_Fig7_ESM.png]

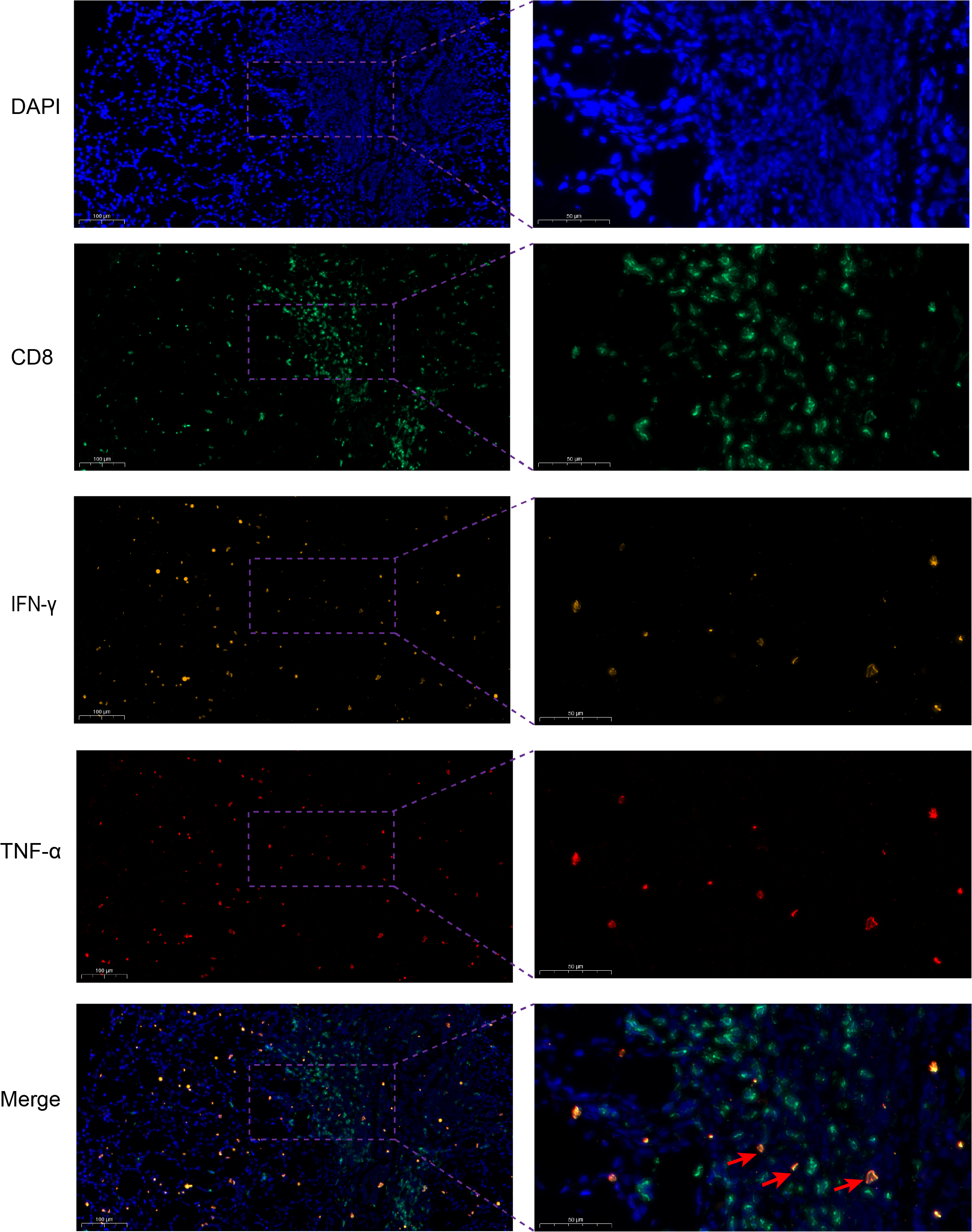

Supplement: Supplementary file 3 — The expression of CD8, IFN-γ and TNF-α in lymphocytic foci of LSG tissues. The molecules of CD8 (green), IFN-γ (orange) and TNF-α (red) were detected by immunofluorescence in the paraffin-embedded sections of LSG tissue of a pSS patient with focus score 2.9, and DAPI was used to counterstain the nuclei. Right magnified pictures show only a few CD8+ T cells of lymphocytic foci were positive for IFN-γ and TNF-α which are directed by red arrows in the Merge picture. Left scale bars = 100 μm, right scale bars = 50 μm. (PNG 2695 kb) [file 10067_2022_6491_Fig8_ESM.png]

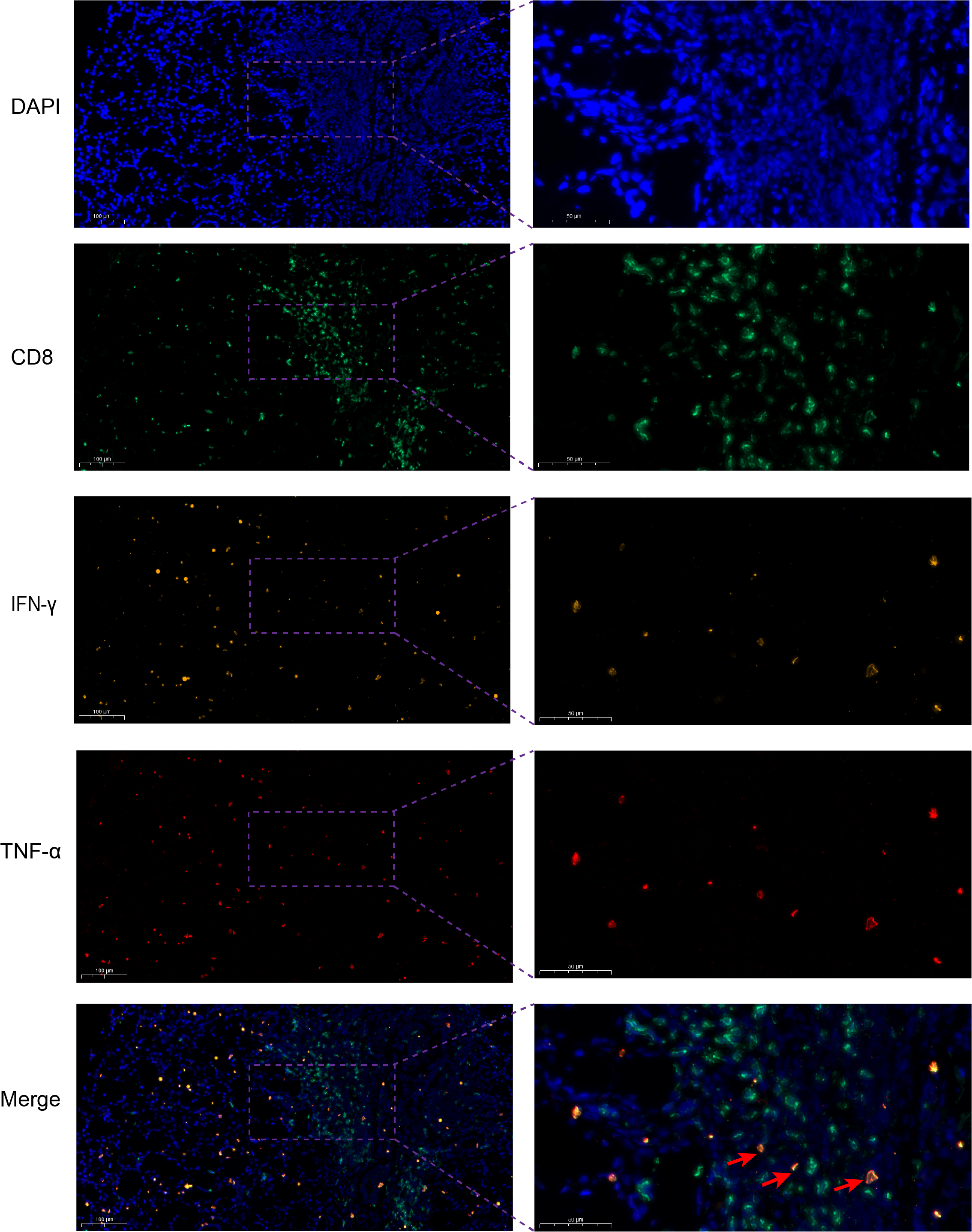

Supplement: Supplementary file 4 — High Resolution Image (TIF 22841 kb) [file 10067_2022_6491_MOESM2_ESM.tif]
